# Lung function and systemic inflammation associated with short-term air pollution exposure in chronic obstructive pulmonary disease patients in Beijing, China

**DOI:** 10.1186/s12940-020-0568-1

**Published:** 2020-01-30

**Authors:** Nannan Gao, Wenshuai Xu, Jiadong Ji, Yanli Yang, Shao-Ting Wang, Jun Wang, Xiang Chen, Shuzhen Meng, Xinlun Tian, Kai-Feng Xu

**Affiliations:** 1 Department of Pulmonary and Critical Care Medicine, Peking Union Medical College Hospital, Peking Union Medical College, Chinese Academy of Medical Sciences, No.1 Wangfujing Hutong, Beijing, 100730 China; 2 School of Statistics, Shandong University of Finance and Economics, Jinan, 250014 China; 3 Beijing Key Laboratory of Precision Medicine for Diagnosis and Treatment on Allergic Diseases, Beijing, 100730 China

**Keywords:** Air pollution, COPD, Lung function, Systemic cytokines

## Abstract

**Background:**

Exposure to air pollution is associated with chronic obstructive pulmonary disease (COPD). However, findings on the effects of air pollution on lung function and systemic inflammation in Chinese COPD patients are inconsistent and scarce. This study aims to evaluate the effects of ambient air pollution on lung function parameters and serum cytokine levels in a COPD cohort in Beijing, China.

**Methods:**

We enrolled COPD participants on a rolling basis from December 2015 to September 2017 in Beijing, China. Follow-ups were performed every 3 months for each participant. Serum levels of 20 cytokines were detected every 6 months. Hourly ambient pollutant levels over the same periods were obtained from 35 monitoring stations across Beijing. Geocoded residential addresses of the participants were used to estimate daily mean pollution exposures. A linear mixed-effect model was applied to explore the effects of air pollutants on health in the first-year of follow-up.

**Results:**

A total of 84 COPD patients were enrolled at baseline. Of those, 75 COPD patients completed the first-year of follow-up. We found adverse cumulative effects of particulate matter less than 2.5 μm in aerodynamic diameter (PM_2.5_), nitrogen dioxide (NO_2_), sulfur dioxide (SO_2_) and carbon monoxide (CO) on the forced vital capacity % predicted (FVC % pred) in patients with COPD. Further analyses illustrated that among COPD patients, air pollution exposure was associated with reduced levels of serum eotaxin, interleukin 4 (IL-4) and IL-13 and was correlated with increased serum IL-2, IL-12, IL-17A, interferon γ (IFNγ), monocyte displacing protein 1 (MCP-1) and soluble CD40 ligand (sCD40L).

**Conclusion:**

Acute exposures to PM_2.5_, NO_2_, SO_2_ and CO were associated with a reduction in FVC % pred in COPD patients. Furthermore, short-term exposure to air pollutants increased systemic inflammation in COPD patients; this may be attributed to increased Th1 and Th17 cytokines and decreased Th2 cytokines.

## Background

Industrialization and urbanization have caused air pollution issues in China; these issues have challenged public health and posed a substantial economic burden [1, 2]. Many epidemiological studies have illustrated that air pollution exposure is correlated with an increased risk of hospitalization and mortality in individuals with chronic obstructive pulmonary disease (COPD) [3–5], which is characterized by irreversible airflow limitation and has a high prevalence in China [6, 7]. As a noninvasive and readily available test, spirometry is the most reproducible and objective measurement tool to diagnose COPD and assess disease severity in any healthcare setting. However, the existing results of the effects of air pollution on lung function are inconsistent, and studies conducted in China have been limited [8].

Several panel studies from Western countries found a negative association between increased levels of particulate matter with an aerodynamic diameter less than 10 μm (PM_10_), particulate matter less than 2.5 μm in aerodynamic diameter (PM_2.5_) and forced vital capacity (FVC) [9] or forced expiratory volume in one second (FEV_1_) [10]. Ni et al. observed that increased ambient PM_10_ and PM_2.5_ levels were associated with decreased FVC in 33 Chinese COPD patients [11]. However, some epidemiological studies found no correlation between air pollution exposure and lung function parameters [12–14]. It is believed that the discrepancies among studies may be ascribed to heterogeneity in the study designs, relatively small sample sizes (less than 40 participants), short follow-up periods (ranging from 67 days to 6 months), spatial and temporal variability and complex local meteorological conditions.

Published studies have indicated that air pollution may cause adverse effects on health via oxidative stress [15, 16], inflammatory response [17] and immune regulation [18]. Pulmonary inflammation is enhanced with exposure to air pollutants [19], especially in COPD patients. Compared with healthy human bronchial epithelial cells, COPD bronchial epithelial cells exhibit increased responsiveness to repeated exposure to PM and decreased capacity to metabolize toxins [20]. Additionally, some studies revealed that air pollution may influence systemic inflammation, which can be reflected by serum cytokine levels and white blood cell (WBC) counts [21]. Dubowsky SD et al. found positive associations between PM_2.5_ and interleukin-6 (IL-6), C-reactive protein (CRP) and WBC counts, with the stronger correlations in people with diabetes, obesity and hypertension [22].

Studies focusing on the effects of air pollution on systemic inflammation in COPD patients are scarce. Macrophages, neutrophils, eosinophils and T helper (Th) cells play important roles in biological inflammatory and immune responses by generating cytokines that act as regulators and effectors and can be identified by high-throughput screening. Therefore, we designed a longitudinal study in Beijing, China, to explore the effects of air pollution on lung function and systemic inflammation in COPD patients. This study may contribute to elucidating the underlying mechanism and identifying the systemic biomarkers involved in the relationship between health effects and air pollution.

## Materials and methods

### Study design and population

We performed a 2-year observational cohort study to explore the effects of air pollution on COPD patients in Beijing, China. All subjects aged 18 to 75 years and residing in Beijing for at least 1 year were eligible to participate. Subjects were enrolled on a rolling basis from December 2015 to September 2017 at Peking Union Medical College Hospital and in the community. Recruitment and follow-ups were processed simultaneously. All included participants completed the first-year follow-up until September 2018. Only 6 COPD patients completed the 2-year follow-up in September 2018. Therefore, the analysis in this study was restricted to data obtained from the first-year follow-up.

The inclusion criteria for COPD patients were physician-diagnosed COPD and a postbronchodilator FEV_1_/FVC < 0.70 according to the Global Initiatives for Chronic Obstructive Lung Disease [23]. Patients with asthma/COPD overlap were excluded according to the Global Initiative for Asthma guidelines [24]. The exclusion criteria are shown in Additional file 1.

Each participant was scheduled to visit the Peking Union Medical College Hospital at three-month intervals. Data on demographic and social characteristics, disease duration, and medication usage for COPD and other existing comorbidities were surveyed via questionnaires at baseline. Doctors inquired about and recorded data on the acute exacerbation of COPD (AECOPD) within the preceding 3 months.

### Lung function measurements

Spirometry was performed at each visit by a professional technician according to the American Thoracic Society/European Respiratory Society standards [25]. The absolute values and the percent predicted FEV_1_ (FEV_1_% pred) and FVC (FVC % pred) were measured.

### Blood sample collection and serum cytokine detection

All visits were scheduled in the morning, and fasting blood samples were collected at each visit. Serum samples were frozen and stored at − 80 °C in the Peking Union Medical Hospital Biobank. At the baseline (1st visit) and 5th visit, blood cell counts, liver function, renal function and lipid profiles were measured at the clinical laboratory of Peking Union Medical College Hospital.

As shown in Additional file 2: Figure S1, we selected three time points to detect cytokine levels: the 1st visit, 3rd visit, 5th visit. Thirty COPD participants (with a total of 90 samples (30*3)) were selected from our cohort to measure cytokine levels using a MILLIPLEX® MAP human cytokine/chemokine magnetic bead panel kit (Merck Millipore Corporation, USA). Each individual serum sample was detected in duplicate. Subjects whose serum were subjected to cytokine detection were not current smokers, and underwent spirometry tests and blood sampling at each visit. In addition, COPD patients without comorbidities had priority for inclusion, followed by those with only dyslipidemia and finally by those with only hypertension.

The following cytokines were detected: IL-1β, IL-2, IL-4, IL-5, IL-6, IL-8, IL-10, IL-12P70, IL-13, IL-17A, tumor necrosis factor α (TNFα), interferon γ (IFNγ), vascular endothelial growth factor A (VEGF-A), monocyte displacing protein 1 (MCP-1), interferon gamma-induced protein (IP-10), granulocyte-macrophage colony stimulating factor (GM-CSF), soluble CD40 ligand (sCD40L), macrophage inflammatory protein (MIP-1α), MIP-1β and eotaxin.

### Air pollution and meteorological data

**Fig. 1:**
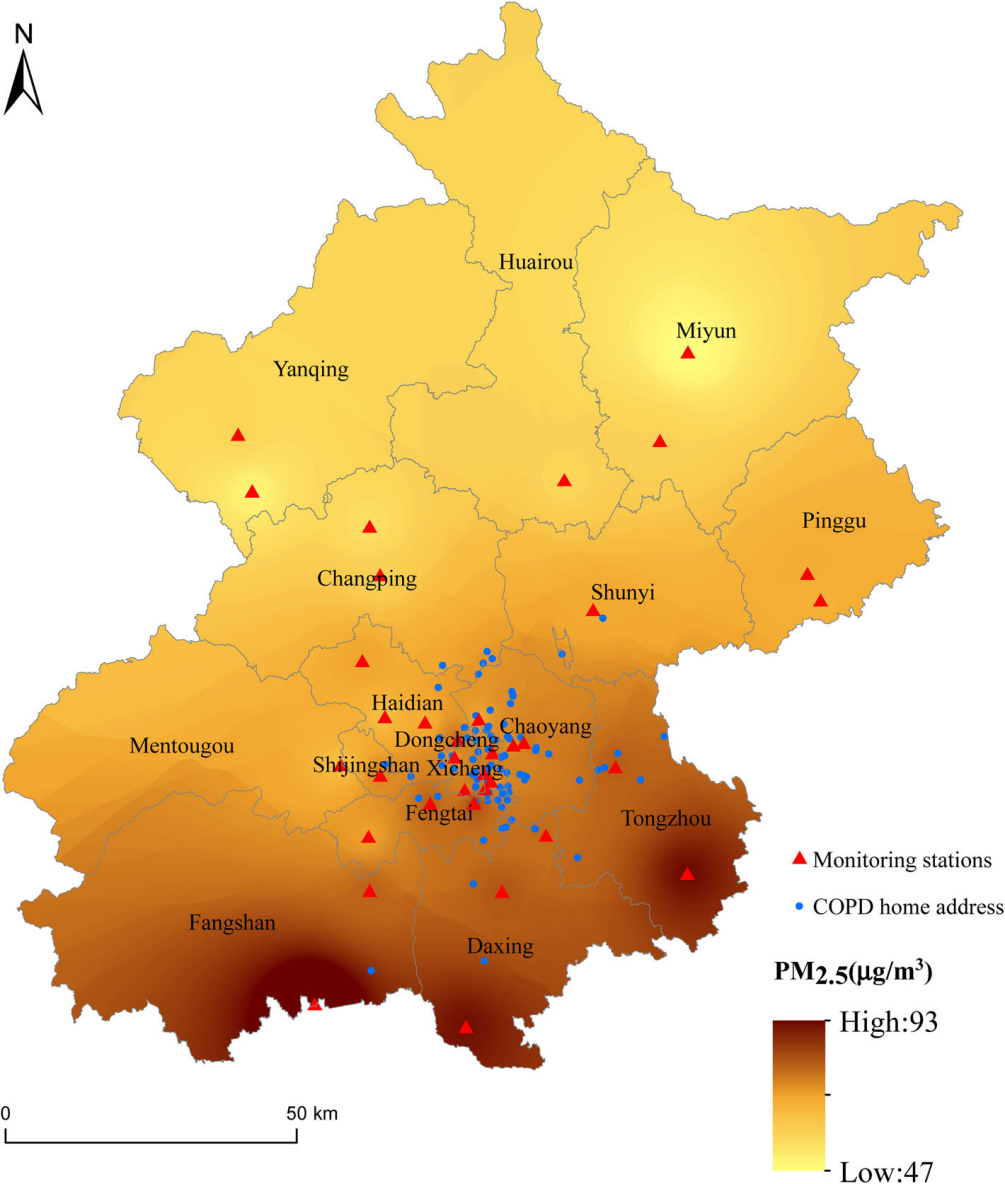
Distributions of participant home addresses and monitoring sites during the study period in Beijing

Data regarding PM_2.5_, PM_10_, nitrogen dioxide (NO_2_), sulfur dioxide (SO_2_), carbon monoxide (CO) and ozone (O_3_) were collected. Hourly air pollutant concentrations at 35 monitoring stations throughout Beijing were obtained from the Beijing Municipal Environmental Protection Bureau (http://www.bjepb.gov.cn/). Daily estimates of pollutants at each monitoring station were calculated as the 24-h mean concentrations for the corresponding station. The residential addresses of each subject and the monitoring sites were geocoded (Fig. 1). The exposed pollutant levels for each participant were approximated using the daily estimate of the monitoring site nearest to the participant’s residential address. In this study, the mean distance from the residential address to the monitoring station was 3.98 km. In our dataset, the proportion of missing daily pollutant levels was 0.45%. For the missing data points, the air pollutant levels were calculated as the city daily estimates. Daily mean temperature and relative humidity in Beijing were collected from the China Meteorological Data Sharing Service System (http://data.cma.cn/).

### Statistical analysis

A linear mixed-effect (LME) model was applied to explore the short-term effects of air pollution on lung function and cytokine levels. By including a random intercept for each subject, the LME model has the advantage of accounting for the correlation between repeated measurements collected per person over time [22]. The model was adjusted for age, sex, marital status, educational level, smoking history, body mass index (BMI) and daily temperature and humidity. The lag days ranged from 0 days (the current day) to the preceding 7 days in the LME model. The single-day lag effect (lag N, *N* = 0,1, … 7) and cumulative lag effect (lag 0 N) were evaluated.

Analyses were performed with R Statistical Software using the ‘lmerTest’ package. Significance was considered at the *p* < 0.05 level. The results were reported as changes with 95% confidence intervals (95% CIs) in pulmonary function and cytokine levels for each standard deviation (SD) increment of air pollutant.

## Results

In our study, 84 COPD patients were eligible for enrollment. The study period ranged from December 2015 to September 2018. As described in the flow chart, 9 patients withdrew from this study after the 1-year follow-up (Additional file 2: Figure S1). There were 9 missing spirometry tests. Table 1 summarizes the baseline characteristics of the COPD patients. Most enrolled participants were male (90.5%), and 22 COPD patients were current smokers.

**Table 1:** Demographic and clinical characteristics of the COPD participants at baseline^*^

	COPD (*n* = 84)
Age (years)	63.9 ± 6.3
Sex (male)	76 (90.5)
Smoking	
Never smoker	9 (10.7)
Former smoker	53 (63.1)
Current smoker	22 (26.2)
FEV_1_ (L)	1.71 ± 0.66
FEV_1_% pred (%)	58.4 ± 19.5
FVC (L)	3.32 ± 0.92
FVC % pred (%)	88.1 ± 15.9
FEV_1_/FVC (%)	51.7 ± 12.4
White blood cell (10^9^ /L)	6.77 ± 1.42
Neutrophil (10^9^ /L)	4.12 ± 1.14
Eosinophil (10^9^ /L)	0.20 ± 0.13
Comorbidity	
Hypertension	26 (31.0)
Diabetes mellitus	8 (9.5)
Coronary heart disease	12 (14.3)
Dyslipidemia	12 (14.3)

Notes: *Data are presented as the mean ± SD or No. (%)

Abbreviations: *COPD* chronic obstructive pulmonary disease, *FEV*_1_ forced expiratory volume in one second, *FVC* forced vital capacity, *%pred* % predicted, *SD* standard deviation

**Fig. 2:**
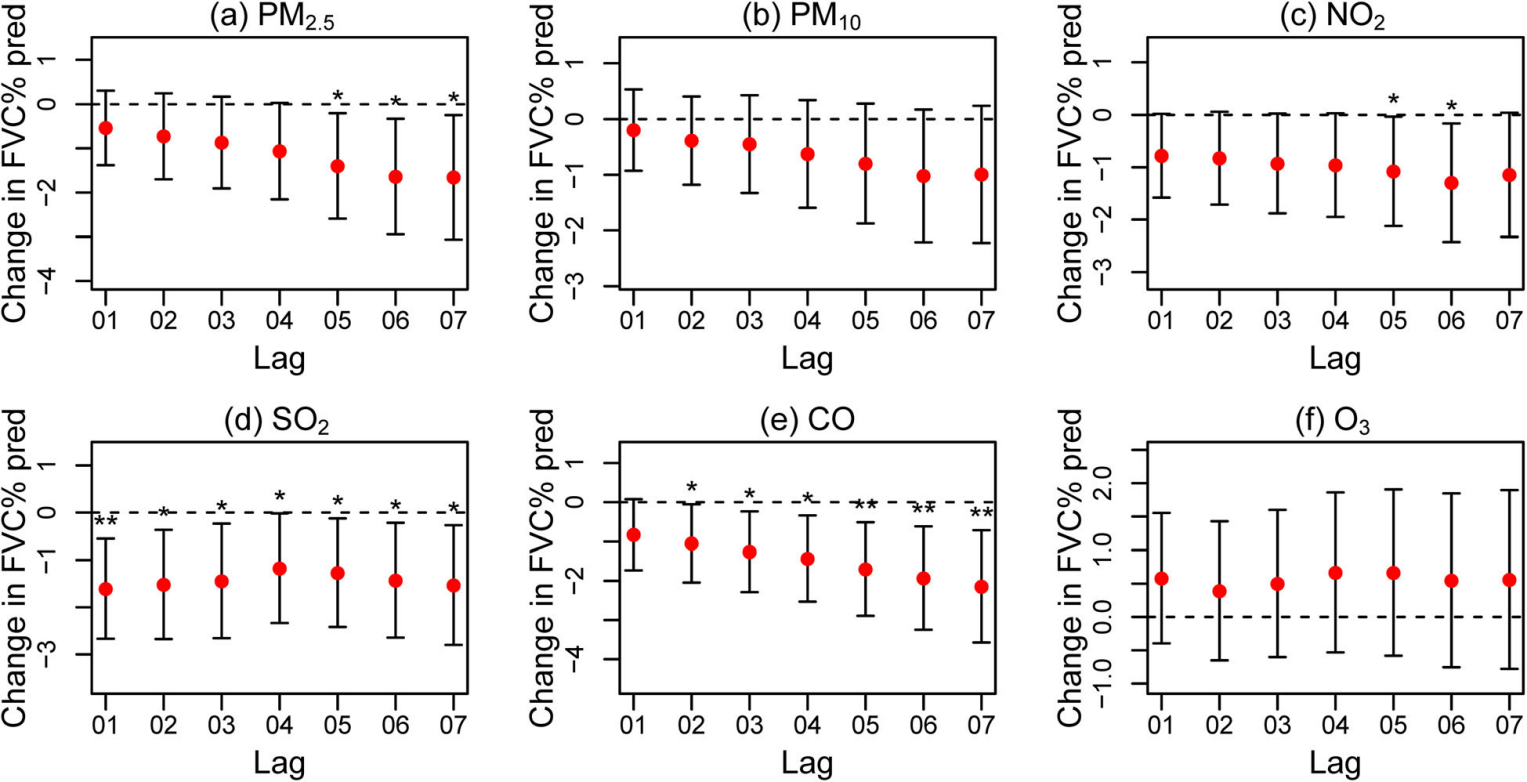
Changes in FVC % pred in COPD patients with a 1 SD increase in PM_2.5_ **a**, PM_10_ **b**, NO_2_ **c**, SO_2_ **d**, CO **e** and O_3_ **f** levels using the cumulative lag model. Notes: Error bars indicate 95% CIs. *p < 0.05; **p < 0.01

Figure 2 and Additional file 2: Figure S2 outlines the short-term effects of air pollutants on FVC % pred in COPD patients. In the single-day lag model, the increases in PM_2.5_, PM_10_, NO_2_, SO_2_ and CO were associated with decreases in FVC % pred (Additional file 2: Figure S2). In the multiday lag model, increased PM_2.5_, NO_2_, SO_2_ and CO levels were correlated with reduced FVC % pred in COPD patients (Fig. 2). We did not observe a similar association between FEV_1_, FEV_1_% pred and increased pollutant levels in COPD patients.

**Fig. 3:**
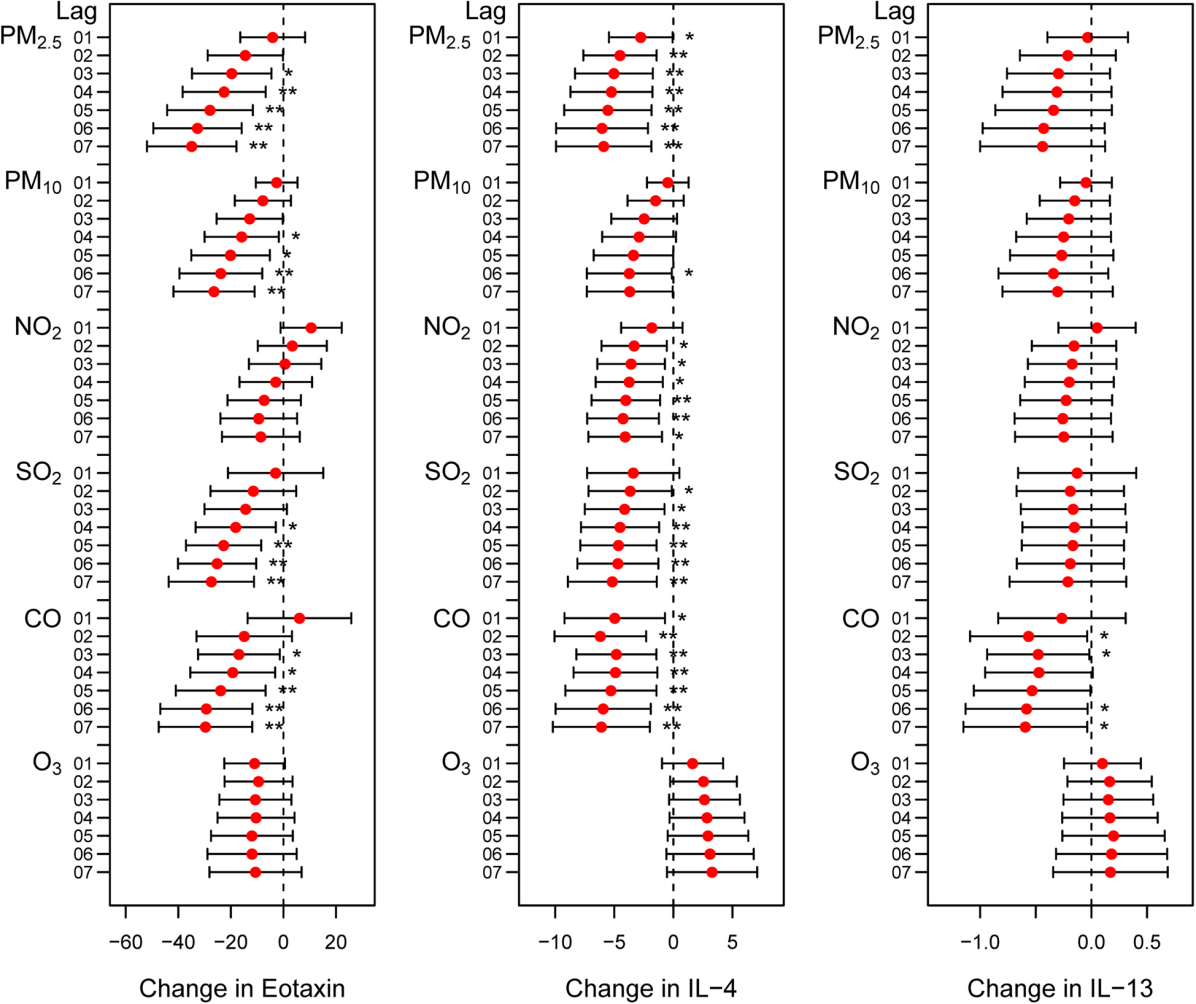
Changes in eotaxin, IL-4 and IL-13 levels in COPD patients with a 1 SD increase in air pollutant levels using the cumulative lag model. Notes: Error bars indicate 95% CIs. *p < 0.05; **p < 0.01

The baseline characteristics of serum cytokines and pollutant exposures for COPD patients are depicted in Additional file 2: Table S1 and Additional file 2: Table S2. Effects of air pollution on cytokine levels are summarized in Additional file 2: Table S3. We observed that circulating levels of eotaxin decreased with increased PM_2.5_, PM_10_, SO_2_ and CO levels in COPD patients (Fig. 3 and Additional file 2: Figure S3). Significant reductions in IL-4 were associated with increased exposures to PM_2.5_, PM_10_, NO_2_, SO_2_ and CO (Fig. 3 and Additional file 2: Figure S3). These correlations increased with increased moving averages. Similar associations were observed between IL-13 and CO (Fig. 3 and Additional file 2: Figure S3).

**Fig. 4:**
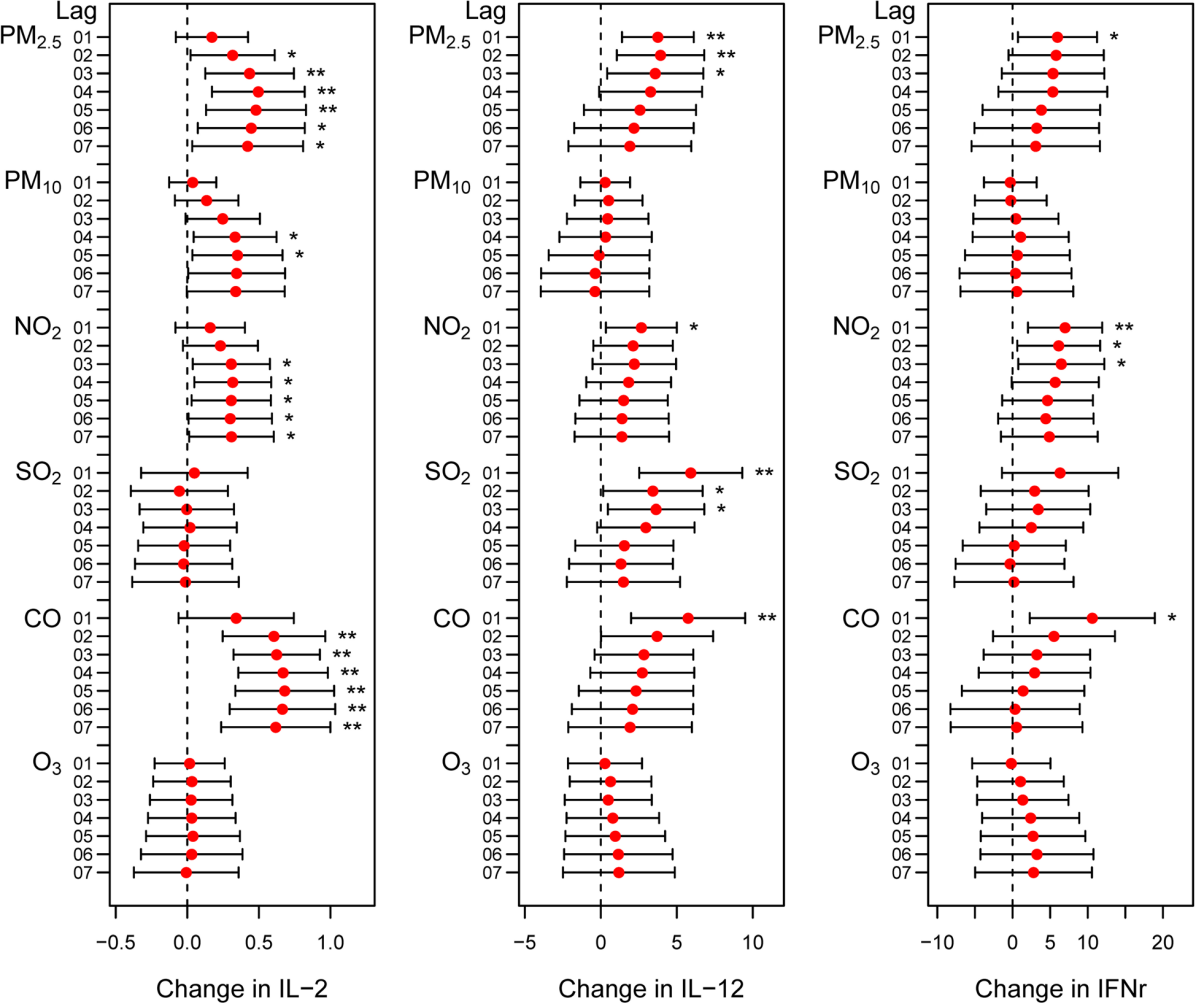
Changes in IL-2, IL-12 and IFNγ levels in COPD patients with a 1 SD increase in air pollutant levels using the cumulative lag model. Notes: Error bars indicate 95% CIs. *p < 0.05; **p < 0.01

**Fig. 5:**
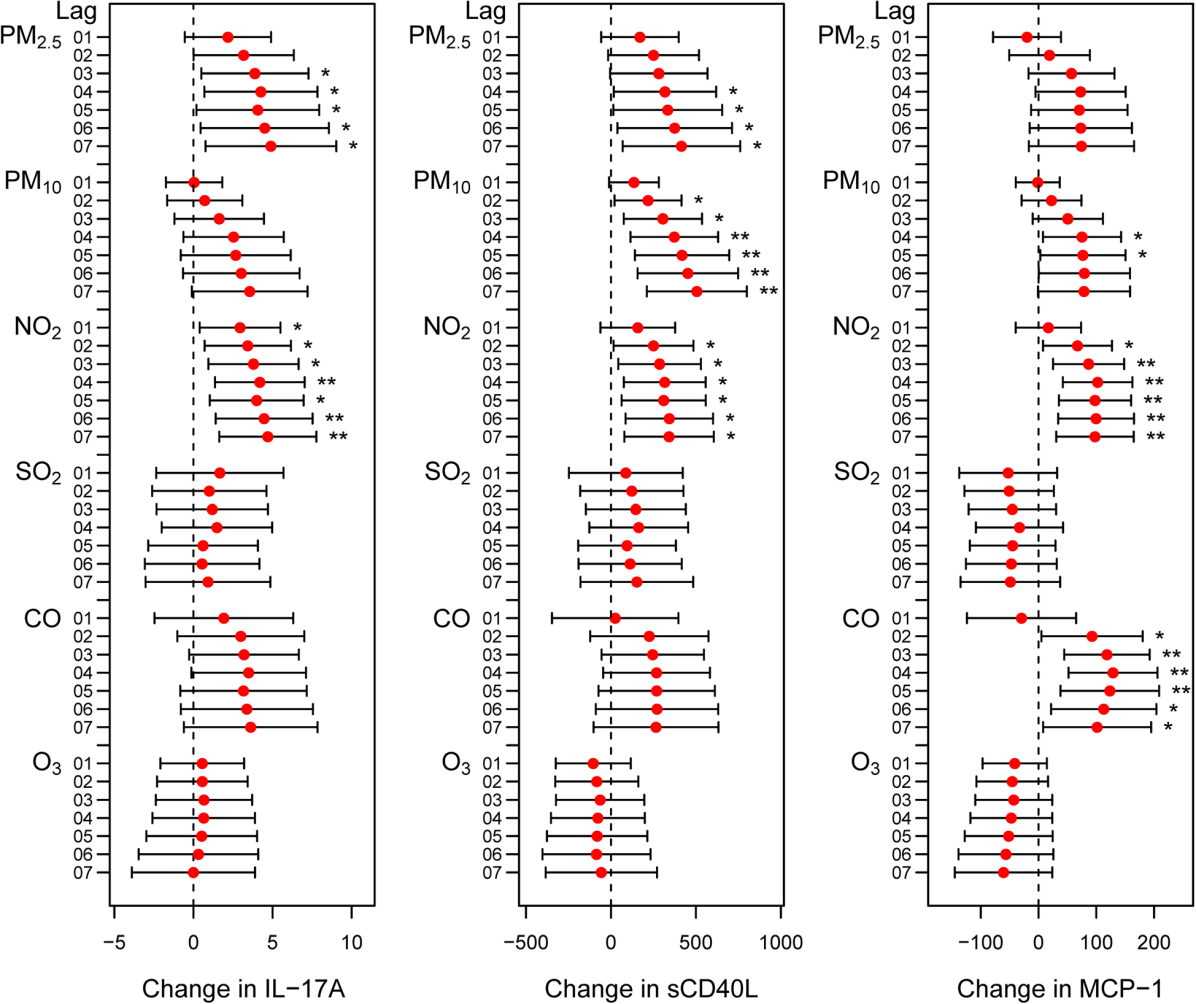
Changes in IL-17A, MCP-1 and sCD40L levels in COPD patients with a 1 SD increase in air pollutant levels using the cumulative lag model. Notes: Error bars indicate 95% CIs. *p < 0.05; **p < 0.01

Figure 4 and Additional file 2: Figure S4 shows the relationships between air pollutant levels and IL-2, IL-12 and IFNγ in COPD patients. Circulating IL-2 levels increased with increased PM_2.5_, PM_10_, NO_2_ and CO exposure (Fig. 4 and Additional file 2: Figure S4). The serum IL-12 levels of COPD patients increased with increasing PM_2.5_, SO_2_, NO_2_ and CO concentrations (Fig. 4 and Additional file 2: Figure S4). IFNγ was positively associated with the levels of PM_2.5_, NO_2_ and CO (Fig. 4 and Additional file 2: Figure S4). In addition, IL-17A increased with increased exposures to PM_2.5_ and NO_2_ (Fig. 5 and Additional file 2: Figure S5). Moreover, serum sCD40L increased with increasing PM_2.5_, PM_10_ and NO_2_ levels (Fig. 5 and Additional file 2: Figure S5). For MCP-1, similar correlations were observed with increased exposure to PM_10_, NO_2_ and CO (Fig. 5 and Additional file 2: Figure S5).

**Fig. 6:**
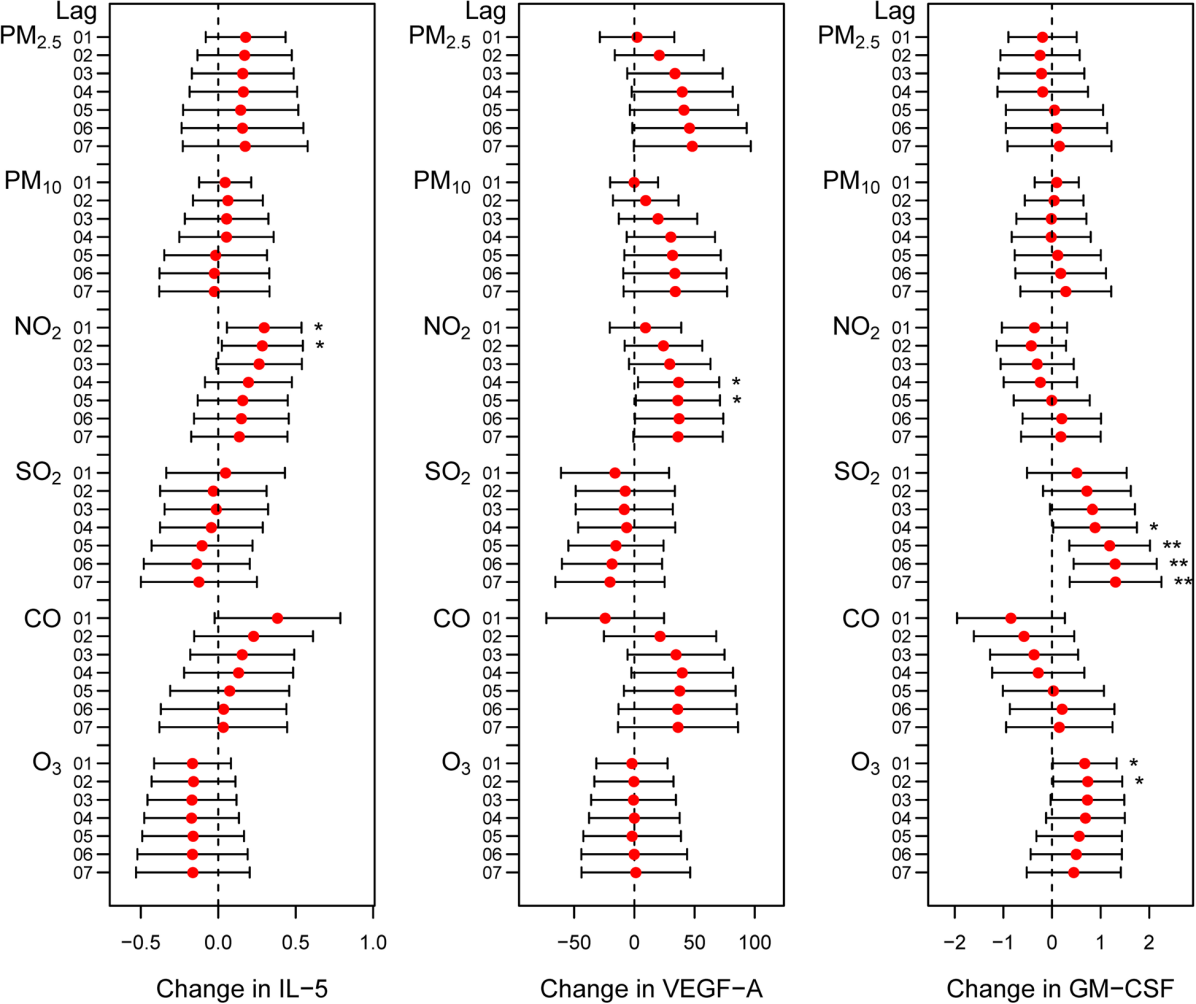
Changes in IL-5, VEGF-A and GM-CSF levels in COPD patients with a 1 SD increase in air pollutant levels using the cumulative lag model. Notes: Error bars indicate 95% CIs. *p < 0.05; **p < 0.01

Increasing serum IL-5 levels were correlated with NO_2_ levels (Fig. 6 and Additional file 2: Figure S6). VEGF-A levels were increased with NO_2_ levels (Fig. 6 and Additional file 2: Figure S6). Increasing GM-CSF was associated with SO_2_ and O_3_ exposures (Fig. 6 and Additional file 2: Figure S6). Correlations between air pollutants, lung function measurements and cytokines are presented in Additional file 2: Table S4, Table S5 and Table S6. There are close correlations between air pollutants, lung function parameters and several biomarker levels.

## Discussion

Our study illustrated that short-term exposure to PM_2.5_, NO_2_, SO_2_ and CO was associated with decreased FVC % pred in COPD patients. Furthermore, we found evidence of associations between air pollution and decreased levels of circulating eotaxin, IL-4 and IL-13 and increased levels of IL-2, IL-12, IL-17A, IFNγ, MCP-1 and sCD40L in COPD patients.

Several previous epidemiological studies reported inconsistent associations between air pollution and lung function in COPD populations. In our study, we confirmed the negative effects of multiple air pollutants on FVC % pred but not on FEV_1_ in COPD patients. These effects were consistent with a study conducted in America, which found that ambient PM_2.5_ was only associated with a decrease in FVC and not FEV_1_ in among individuals with COPD [26]. Ni et al. found that interquartile range (IQR) increases in outdoor PM_2.5_ and PM_10_ were associated with a 3.3% and a 2.1% reduction in FVC, respectively [11]. A similar correlation was only found between ambient PM_2.5_ and FEV_1_ [11]. However, some studies found no effects of PM exposure on lung function parameters in COPD patients [12, 27].

Enhanced chronic inflammatory responses and abnormal immune responses play important roles in COPD pathogenesis and progression [28]. Imbalance in T cell subsets has been implicated in the immune and inflammatory responses of COPD [29, 30]. In addition to the airway inflammatory response, several studies illustrated that COPD progression was also associated with systemic inflammation marked by increased WBC counts and TNF-ɑ and IL-6 levels [31]. Our study showed that COPD patients had decreased circulating IL-4, IL-13 and eotaxin levels when exposed to air pollution. Th2 cells are anti-inflammatory cells that produce IL-4 and IL-13, which can stimulate the expression of eotaxin generated by eosinophils, macrophages and alveolar epithelial cells [32]. Therefore, there is a synergistic effect between IL-4, IL-13 and eotaxin.

Moreover, we observed an association between serum IL-2, IL-12, IFNγ and IL-17A levels and air pollution exposure. As important proinflammatory cytokines, IL-2, IL-12 and IFNγ are generated by Th1 cells, and IL-17A is secreted by Th17 cells [33]. The collective pattern of changes in serum cytokines may be indicative of increased systemic inflammation in COPD patients exposed to ambient air pollution due to aggravation of the Th1/Th2 and Th17 imbalance. Our results were consistent with the findings of Gu et al. who found that Th1, Th17, IFN-γ and IL-17 levels were increased in association with air pollution in COPD mice, while IL-4, IL-10, Th2 and regulatory T cells (Tregs) were significantly decreased compared with levels in the healthy group [34]. Therefore, PM_2.5_ exposure aggravates Th1- and Th17-mediated immune disorders [34].

We also observed that short-term exposure to PM_2.5_, PM_10_ and NO_2_ increased the expression of circulating MCP-1 in COPD patients; this increased circulation could promote inflammatory responses in COPD patients by inducing the accumulation of monocytes and macrophages [35]. Additionally, MCP-1 also plays a role in regulating Th cell differentiation in vivo [36]. sCD40L is released from activated platelets and T cells with proinflammatory and prothrombotic characteristics. The existing evidence regarding PM and sCD40L mainly focuses on cardiovascular disease [37]. However, some studies have indicated that sCD40L plays a contributing role in pulmonary emphysema [38].

There are several limitations in our study. First, we used ambient air pollutant levels from monitoring sites to estimate personal exposure, which may induce estimation bias. Further research utilizing personal exposure devices may be helpful to overcome this bias in the future. Second, multiple testing was not corrected in this study. Given the high level of correlations between tests, it is difficult to correct the *p* value or control the false discovery rate. Bonferroni correction is the common method used to correct the p value in the multiple testing method, but it may cause false negative results. Therefore, we did not apply it in this research. Third, our model was adjusted for age, sex, marital status, educational level, smoking history, BMI, temperature and humidity as potential confounders from published references. However, it is difficult to confirm that these factors are “real” confounders. For example, Fuertes E et al. applied adjustments for height and weight instead of BMI as confounding factors to elucidate the associations between physical activity and lung function [39].

## Conclusion

In summary, short-term exposure to PM_2.5_, NO_2_, SO_2_ and CO may decrease the FVC % pred in COPD patients. The characteristic patterns of changes in cytokines in the COPD patients reported in our research suggested that exposure to air pollutants may enhance systemic inflammation in COPD patients by increasing Th1 and Th17 cytokines and decreasing Th2 cytokines. These findings provide new insights into the potential mechanisms by which air pollution triggers or exacerbates COPD.

## Supplementary information

**Additional file 1.** The exclusion criteria in the study

**Additional file 2: Figure S1.** Study flow chart. **Figure S2.** Changes in FVC % pred in COPD patients with 1 SD increase in PM_2.5_ (a), PM_10_ (b), NO_2_ (c), SO_2_ (d), CO (e) and O_3_ (f) levels using a single-day lag model. **Figure S3.** Changes in eotaxin, IL-4 and IL-13 levels in COPD patients with a 1 SD increase in air pollutant levels using a single-day lag model. **Figure S4.** Changes in IL-2, IL-12 and IFNγ levels in COPD patients with a 1 SD increase in air pollutant levels using a single-day lag model. **Figure S5.** Changes in IL-17A, MCP-1 and sCD40L levels in COPD patients with a 1 SD increase in air pollutant levels using a single-day lag model. **Figure S6.** Changes in IL-5, VEGF-A and GM-CSF levels in COPD patients with a 1 SD increase in air pollutant levels using a single-day lag model. **Table S1.** Characteristics of air pollutant levels for COPD patients in the study. **Table S2.** Baseline serum cytokine levels in COPD cohort. **Table S3.** Summary of the correlations between air pollution exposure and serum cytokine levels. **Table S4.** Correlation coefficients between air pollutants. **Table S5.** Correlation coefficients between lung function. **Table S6.** Spearman correlation coefficients between cytokines

## Data Availability

The datasets generated and/or analyzed during the current study are not publicly available due other analyses are proceeding but are available from the corresponding author on reasonable request.

## Abbreviations

% pred: % predicted
CO: Carbon monoxide
COPD: Chronic obstructive pulmonary disease
FEV_1_: Forced expiratory volume in one second
FVC: Forced vital capacity
GM-CSF: Granulocyte-macrophage colony stimulating factor
IFNγ: Interferon γ
IL: Interleukin
IP-10: Interferon gamma-induced protein
LME: Linear mixed-effect
MCP-1: Monocyte displacing protein 1
MIP: Macrophage inflammatory protein
NO_2_: Nitrogen dioxide
O_3_: Ozone
PM_10_: Particulate matter with an aerodynamic diameter less than 10 μm
PM_2.5_: Particulate matter less than 2.5 μm in aerodynamic diameter
sCD40L: Soluble CD40 ligand
SO_2_: Sulfur dioxide
TNFα: Tumor necrosis factor α
VEGF-A: Vascular endothelial growth factor A
WBC: White blood cell
