# Supplementary material for: Lung function and systemic inflammation associated with short-term air pollution exposure in chronic obstructive pulmonary disease patients in Beijing, China
Source: Environ Health. 2020 Jan 30;19:12. doi: 10.1186/s12940-020-0568-1 (PMC6993429; doi:10.1186/s12940-020-0568-1)
Supplement: Supplementary file 1 — Additional file 1. The exclusion criteria in the study [file 12940_2020_568_MOESM1_ESM.docx]

Lung function and systemic inflammation associated with short-term air pollution exposure in chronic obstructive pulmonary disease patients in Beijing, China

Nannan Gao, Wenshuai Xu, Jiadong Ji, Yanli Yang, Shao-Ting Wang, Jun Wang, Xiang Chen, Shuzhen Meng, Xinlun Tian, Kai-Feng Xu

Exclusion criteria in this study were as followed:

(1) The latest severe acute exacerbation of COPD occurred within 4 weeks.

(2) Any history of acute/chronic respiratory diseases other than COPD, including asthma COPD overlap, asthma, lung cancer and pulmonary infection.

(3) Plasma alanine transaminase (ALT) or aspartate transaminase (AST) greater than 2 times of the upper normal limit, plasma creatinine greater than 1.5 times of the upper normal limit.

(4) Left heart insufficiency, or malignant arrhythmia.

(5) HIV positive.

(6) Acute cerebrovascular events within 3 months, including apoplexy, transient cerebral ischemia and acute coronary syndrome.

(7) Uncured malignant tumors.

(8) Addicted to drug or alcohol, or any history of psychiatric disorders.

(9) Breastfeeding, pregnancy or planning to be pregnant.

(10) Estimated lifetime less than 2 years due to underlying diseases.
