# Supplementary material for: Lung function and systemic inflammation associated with short-term air pollution exposure in chronic obstructive pulmonary disease patients in Beijing, China
Source: Environ Health. 2020 Jan 30;19:12. doi: 10.1186/s12940-020-0568-1 (PMC6993429; doi:10.1186/s12940-020-0568-1)
Supplement: Supplementary file 2 — Additional file 2: Figure S1. Study flow chart. Figure S2. Changes in FVC % pred in COPD patients with 1 SD increase in PM2.5 (a), PM10 (b), NO2 (c), SO2 (d), CO (e) and O3 (f) levels using a single-day lag model. Figure S3. Changes in eotaxin, IL-4 and IL-13 levels in COPD patients with a 1 SD increase in air pollutant levels using a single-day lag model. Figure S4. Changes in IL-2, IL-12 and IFNγ levels in COPD patients with a 1 SD increase in air pollutant levels using a single-day lag model. Figure S5. Changes in IL-17A, MCP-1 and sCD40L levels in COPD patients with a 1 SD increase in air pollutant levels using a single-day lag model. Figure S6. Changes in IL-5, VEGF-A and GM-CSF levels in COPD patients with a 1 SD increase in air pollutant levels using a single-day lag model. Table S1. Characteristics of air pollutant levels for COPD patients in the study. Table S2. Baseline serum cytokine levels in COPD cohort. Table S3. Summary of the correlations between air pollution exposure and serum cytokine levels. Table S4. Correlation coefficients between air pollutants. Table S5. Correlation coefficients between lung function. Table S6. Spearman correlation coefficients between cytokines [file 12940_2020_568_MOESM2_ESM.docx]

Lung function and systemic inflammation associated with short-term air pollution exposure in chronic obstructive pulmonary disease patients in Beijing, China

Nannan Gao, Wenshuai Xu, Jiadong Ji, Yanli Yang, Shao-Ting Wang, Jun Wang, Xiang Chen, Shuzhen Meng, Xinlun Tian, Kai-Feng Xu

**
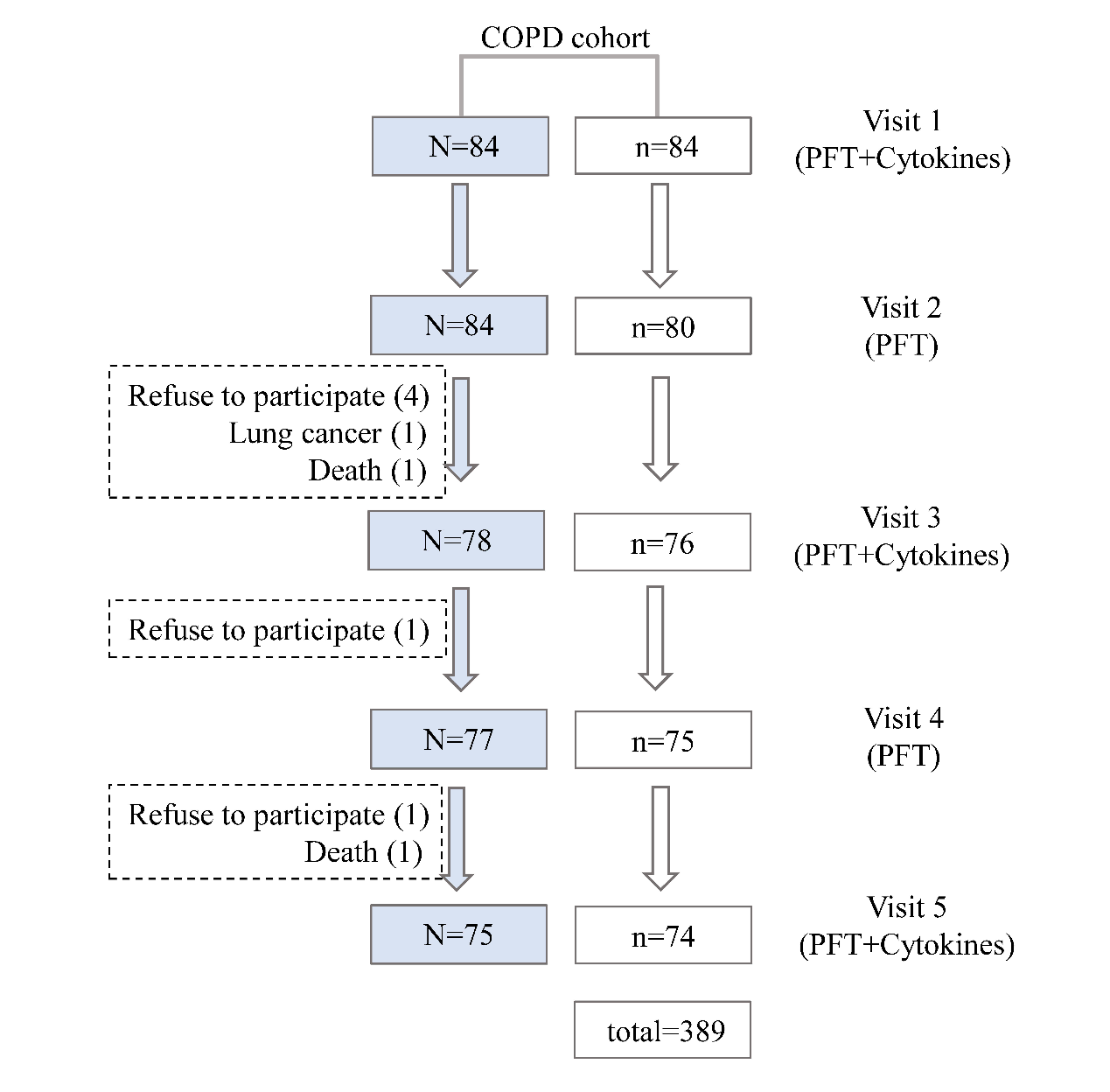
**

**Figure S1 Study flow chart.**

Notes N: number of participants at the corresponding visits for COPD cohort.

n: number of pulmonary function tests (PFT) obtained at the corresponding visits.


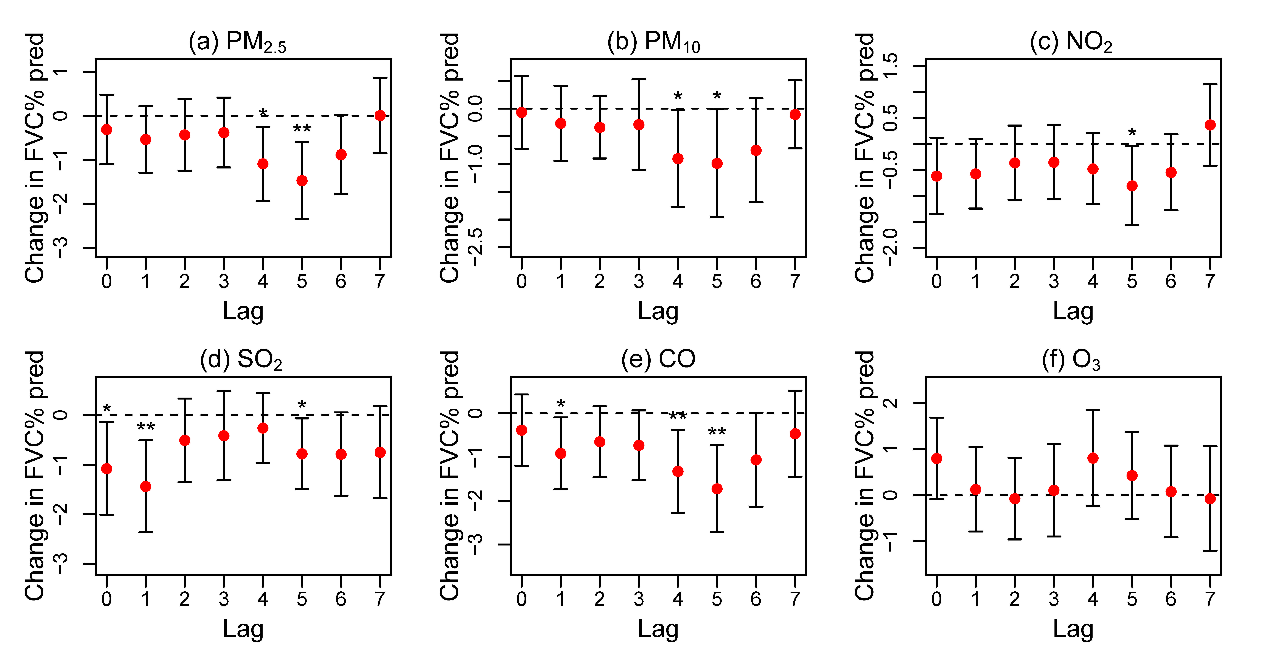


**Figure S2** Changes in FVC % pred in COPD patients with 1 SD increase in PM_2.5_ (a), PM_10_ (b), NO_2_ (c), SO_2_ (d), CO (e) and O_3_ (f) levels using a single-day lag model.

Notes: Error bars indicate 95% CIs. *p<0.05; **p<0.01.


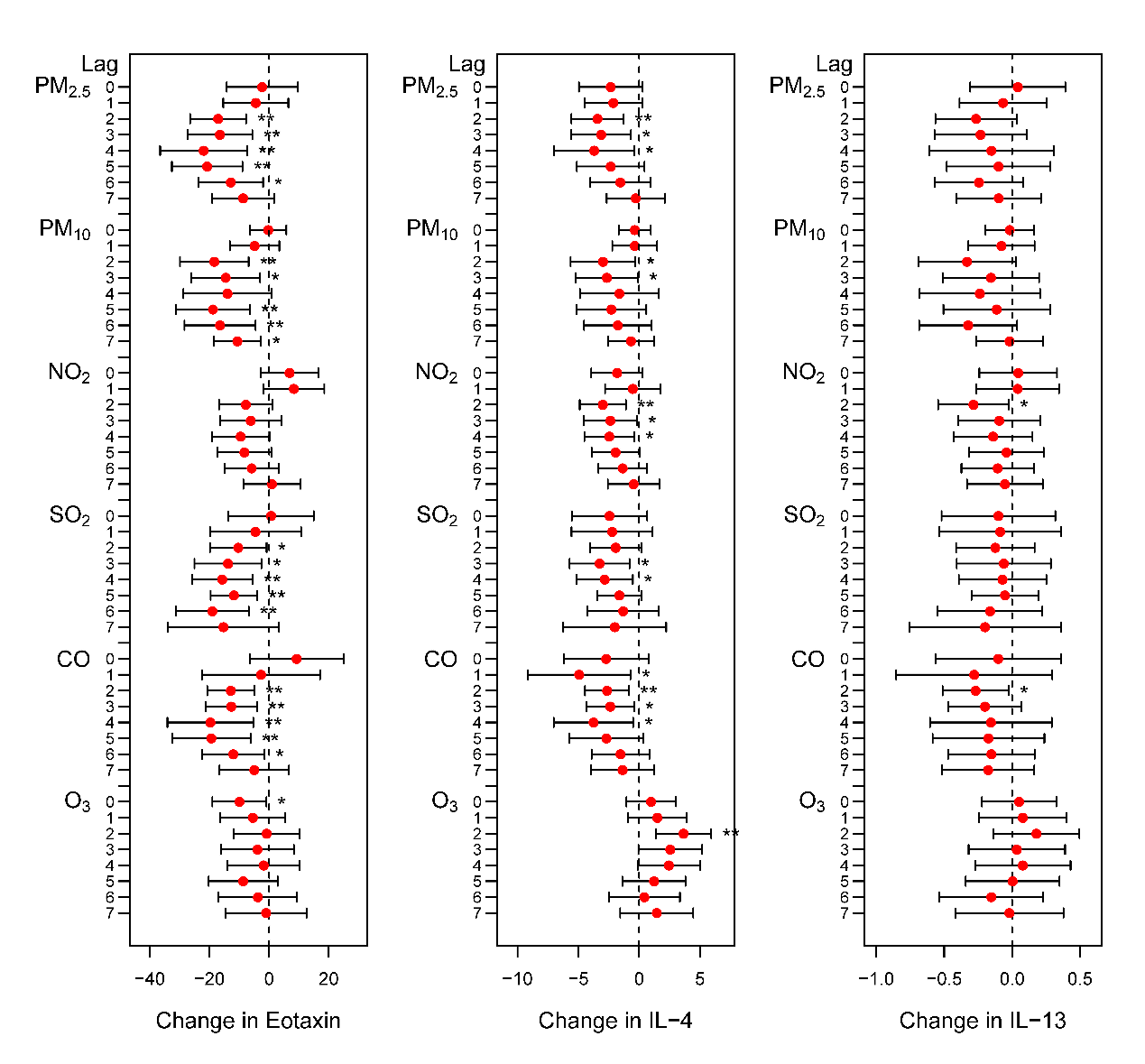


**Figure S3** Changes in eotaxin, IL-4 and IL-13 levels in COPD patients with a 1 SD increase in air pollutant levels using a single-day lag model.

Notes: Error bars indicate 95% CIs. *p<0.05; **p<0.01.


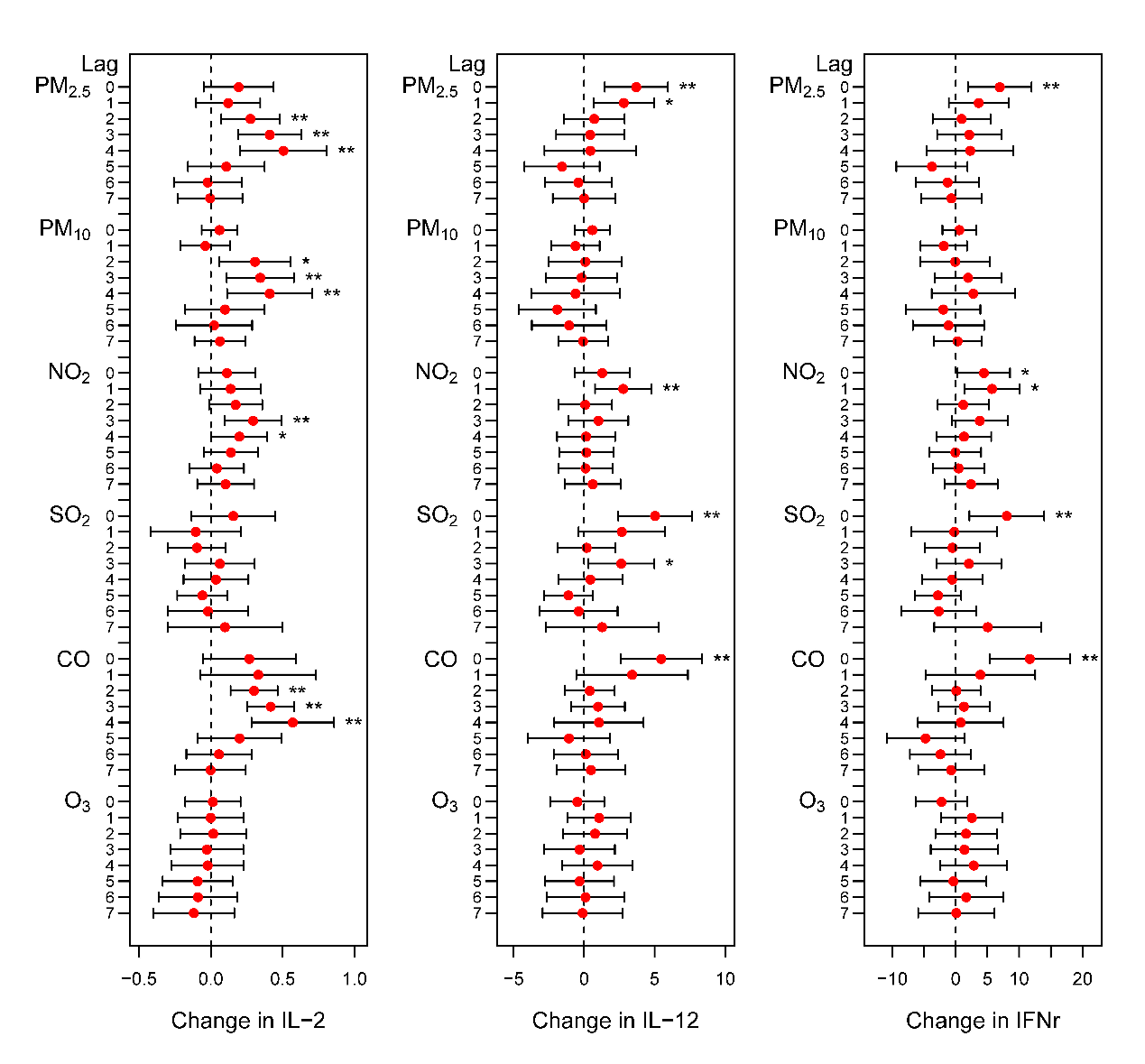


**Figure S4** Changes in IL-2, IL-12 and IFNγ levels in COPD patients with a 1 SD increase in air pollutant levels using a single-day lag model.

Notes: Error bars indicate 95% CIs. *p<0.05; **p<0.01.


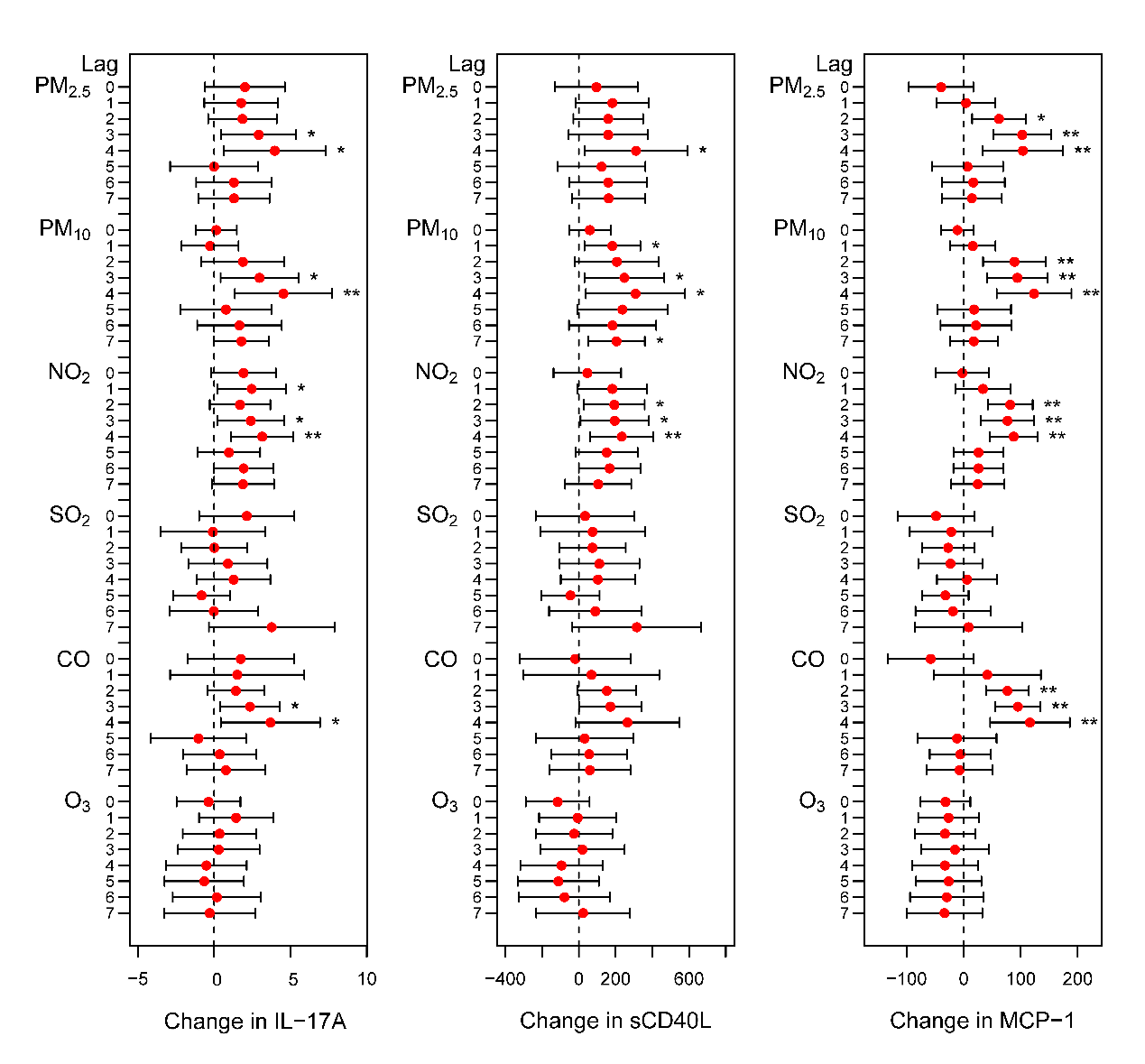


**Figure S5** Changes in IL-17A, MCP-1 and sCD40L levels in COPD patients with a 1 SD increase in air pollutant levels using a single-day lag model.

Notes: Error bars indicate 95% CIs. *p<0.05; **p<0.01.

**
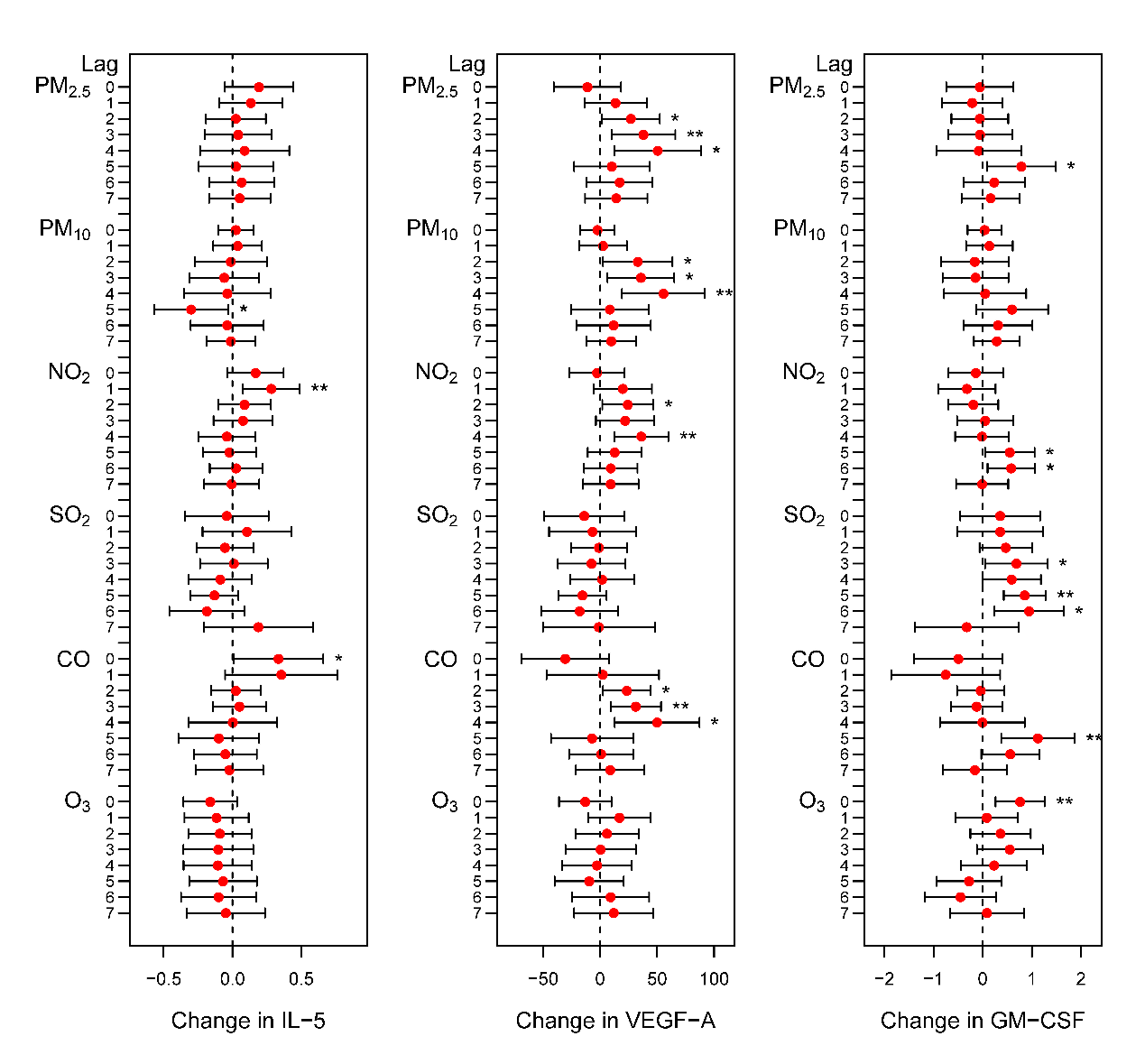
**

**Figure S6** Changes in IL-5, VEGF-A and GM-CSF levels in COPD patients with a 1 SD increase in air pollutant levels using a single-day lag model.

Notes: Error bars indicate 95% CIs. *p<0.05; **p<0.01.

**Table S1** Characteristics of air pollutant levels for COPD patients in the study.

| Air pollutant | Mean±SD^*^ |
| --- | --- |
| PM_10_ (μg/m^3^) | 94.8±86.05 |
| PM_2.5_ (μg/m^3^) | 64.04±66.13 |
| CO (mg/m^3^) | 1.04±0.99 |
| NO_2_(μg/m^3^) | 55.95±25.17 |
| O_3_ (μg/m^3^) | 55.27±44.12 |
| SO_2_ (μg/m^3^) | 8.59±7.40 |

Notes: *mean exposure levels for COPD: firstly, calculated each participant exposure, then averaged all exposures of COPD patients.

Abbreviations: COPD, chronic obstructive pulmonary disease; SD, standard deviation; PM_2.5_, particulate matter less than 2.5μm in aerodynamic diameter; PM_10_, particulate matter less than 10μm in aerodynamic diameter; CO, carbon monoxide; NO_2_, nitrogen dioxide; O_3_, ozone; SO_2_, sulfur dioxide.

**Table S2** Baseline serum cytokine levels in COPD cohort.

| Cytokine | Mean±SD^†^ |
| --- | --- |
| Eotaxin (ng/mL) | 5.10±0.60 |
| GM-CSF (ng/mL) | 2.60±0.23 |
| IFNγ (ng/mL) | 1.88±1.26 |
| IL-10 (ng/mL) | 2.63±0.38 |
| IL-12 (ng/mL) | 2.28±0.63 |
| IL-13 (ng/mL) | 1.66±0.28 |
| sCD40L (ng/mL) | 7.84±0.77 |
| IL-17A (ng/mL) | 1.92±1.36 |
| IL-1β (ng/mL) | 0.70±0.44 |
| IL-2 (ng/mL) | 1.38±0.28 |
| IL-4 (ng/mL) | 3.24±0.39 |
| IL-5 (ng/mL) | 1.18±0.29 |
| IL-6 (ng/mL) | 1.35±0.56 |
| IL-8 (ng/mL) | 2.76±1.00 |
| IP-10 (ng/mL) | 5.70±0.44 |
| MCP-1 (ng/mL) | 6.13±0.46 |
| MIP1α (ng/mL) | 2.61±0.81 |
| MIP1β (ng/mL) | 3.84±0.63 |
| TNFα (ng/mL) | 2.95±0.42 |
| VEGF-A (ng/mL) | 4.93±0.81 |

Notes: † cytokine levels take logarithm.

Abbreviations: COPD, chronic obstructive pulmonary disease; SD, standard deviation; GM-CSF, granulocyte-macrophage colony stimulating factor; IFN, interferon; IL, interleukin; sCD40L, soluble CD40 ligand; IP, interferon gamma-induced protein; MCP-1, monocyte displacing protein 1; MIP, macrophage inflammatory protein; TNF, tumor necrosis factor; vascular endothelial growth factor A, VEGF-A.

**Table S3** Summary of the correlations between air pollution exposure and serum cytokine levels.

|  | COPD population | |  |
| --- | --- | --- | --- |
| Cytokine | effect | exposure |  |
| GM-CSF | ↑ | SO_2_, O_3_ |  |
| IFNγ | ↑ | PM_2.5_, NO_2_, CO |  |
| VEGF-A | ↑ | NO_2_ |  |
| IL-1β | — |  |  |
| IL-2 | ↑ | PM_2.5_, PM_10_, NO_2_, CO |  |
| IL-4 | ↓ | PM_2.5_, PM_10_, NO_2_, SO_2_, CO |  |
| IL-5 | ↑ | NO_2_ |  |
| IL-6 | — |  |  |
| IL-8 | — |  |  |
| IL-10 | — |  |  |
| IL-12P70 | ↑ | PM_2.5_, NO_2_, SO_2_, CO |  |
| IL-13 | ↓ | CO |  |
| IL-17A | ↑ | PM_2.5_, NO_2_ |  |
| IP-10 | — |  |  |
| MCP-1 | ↑ | PM_10_, NO_2_, CO |  |
| MIP-1α | — |  |  |
| sCD40L | ↑ | PM_2.5_, PM_10_, NO_2_ |  |
| TNFα | — |  |  |
| MIP-1β | — |  |  |
| eotaxin | ↓ | PM_2.5_, PM_10_, SO_2_, CO |  |

Notes: ↑ The cytokine level increased with air pollutant exposures; ↓ The cytokine level decreased with air pollutant exposures; — The cytokine level had no association with air pollution levels.

Abbreviations: COPD, chronic obstructive pulmonary disease; CO, carbon monoxide; GM-CSF, granulocyte-macrophage colony stimulating factor; IFN, interferon; IL, interleukin; sCD40L, soluble CD40 ligand; IP, interferon gamma-induced protein; MCP-1, monocyte displacing protein 1; MIP, macrophage inflammatory protein; NO_2_, nitrogen dioxide; O_3_, ozone; PM_2.5_, particulate matter less than 2.5μm in aerodynamic diameter; PM_10_, particulate matter less than 10μm in aerodynamic diameter; SO_2_, sulfur dioxide; TNF, tumor necrosis factor; vascular endothelial growth factor A, VEGF-A.

**Table S4** Correlation coefficients between air pollutants.

|  | PM_10_ | PM_2.5_ | CO | NO_2_ | O_3_ | SO_2_ |
| --- | --- | --- | --- | --- | --- | --- |
| PM_10_ | 1 | 0.82^**^ | 0.66^**^ | 0.66^**^ | -0.12^**^ | 0.47^**^ |
| PM_2.5_ |  | 1 | 0.87^**^ | 0.79^**^ | -0.20^**^ | 0.56^**^ |
| CO |  |  | 1 | 0.83^**^ | -0.38^**^ | 0.61^**^ |
| NO_2_ |  |  |  | 1 | -0.49^**^ | 0.64^**^ |
| O_3_ |  |  |  |  | 1 | -0.35^**^ |
| SO_2_ |  |  |  |  |  | 1 |

Notes: ** p<0.01.

Abbreviations: PM_2.5_, particulate matter less than 2.5μm in aerodynamic diameter; PM_10_, particulate matter less than 10μm in aerodynamic diameter; CO, carbon monoxide; NO_2_, nitrogen dioxide; O_3_, ozone; SO_2_, sulfur dioxide.

**Table S5** Correlation coefficients between lung function.

|  | FEV_1_ | FEV_1_%pred | FVC | FVC%pred |
| --- | --- | --- | --- | --- |
| FEV_1_ | 1 | 0.89^**^ | 0.72^**^ | 0.54^**^ |
| FEV_1_%pred |  | 1 | 0.52^**^ | 0.67^**^ |
| FVC |  |  | 1 | 0.63^**^ |
| FVC%pred |  |  |  | 1 |

Notes: ** p<0.01.

Abbreviations: FEV_1_ % pred: forced expiratory volume in one second % predicted; FVC % pred: forced vital capacity % predicted.

**Table S6** Spearman correlation coefficients between cytokines.

Notes: ** p<0.01; * p<0.05.

Abbreviations: GM-CSF, granulocyte-macrophage colony stimulating factor; IFN, interferon; IL, interleukin; sCD40L, soluble CD40 ligand; IP, interferon gamma-induced protein; MCP-1, monocyte displacing protein 1; MIP, macrophage inflammatory protein; TNF, tumor necrosis factor; vascular endothelial growth factor A, VEGF-A.
